# High-speed alignment optimization of digital optical phase conjugation systems based on autocovariance analysis in conjunction with orthonormal rectangular polynomials

**DOI:** 10.1117/1.JBO.24.3.031004

**Published:** 2018-08-28

**Authors:** Ashton S. Hemphill, Yuecheng Shen, Jeeseong Hwang, Lihong V. Wang

**Affiliations:** a California Institute of Technology, Caltech Optical Imaging Laboratory, Andrew and Peggy Cherng Department of Medical Engineering, Pasadena, California, United States; b California Institute of Technology, Caltech Optical Imaging Laboratory, Department of Electrical Engineering, Pasadena, California, United States; c Washington University in St. Louis, Optical Imaging Laboratory, Department of Biomedical Engineering, St. Louis, Missouri, United States; d National Institute of Standards and Technology, Quantum Electromagnetics Division, Boulder, Colorado, United States

**Keywords:** wavefront shaping, digital optical phase conjugation, time reversal, active or adaptive optics, turbid media

## Abstract

Digital optical phase conjugation (DOPC) enables many optical applications by permitting focusing of light through scattering media. However, DOPC systems require precise alignment of all optical components, particularly of the spatial light modulator (SLM) and camera, in order to accurately record the wavefront and perform playback through the use of time-reversal symmetry. We present a digital compensation technique to optimize the alignment of the SLM in five degrees of freedom, permitting focusing through thick scattering media with a thickness of 5 mm and transport scattering coefficient of $2.5\;{\mathrm{mm}}^{-1}$ while simultaneously improving focal quality, as quantified by the peak-to-background ratio, by several orders of magnitude over an unoptimized alignment.

## 1 Introduction

In biological tissue, optical scattering limits the focusing of light to depths within the optical diffusion limit of ∼1  mm in soft tissue.^1^^,^^2^ This limitation greatly restricts the utility of optical diagnostic and therapeutic techniques, such as optogenetics, microsurgery, optical tweezing, and phototherapy of deep tissue, which require focused light in order to function.^3^^–^^7^ However, optical scattering is, on certain timescales, deterministic, allowing for compensation using several methods.^8^^–^^20^

One such technique, digital optical phase conjugation (DOPC), circumvents the optical diffusion limit through time reversal.^21^^–^^25^ A spatial light modulator (SLM) phase conjugates the optical wavefront incident on the SLM to maximize the light energy reaching a greater depth in a scattering medium. This allows DOPC to focus light through scattering media by causing scattered photons to evolve approximately backward in time as they travel back to their origin.

Because DOPC relies on the principle of time reversal, it is imperative that the symmetry between forward and backward photon propagation be maintained. Time-reversal symmetry relies primarily on two key aspects of the DOPC system. First, to ensure that back propagating photons follow the same paths as in the initial scattering, the scattering medium must be deterministic. The time for which the scattering medium may be assumed to be deterministic, known as the scattering correlation time, determines the time limit for recording and playback by the DOPC system, beyond which focusing is no longer possible.^25^^–^^27^

Second, the phase of the scattered wavefront must be conjugated as accurately as possible to enable back propagation of photons to their origin.^15^ While some inaccuracy may be tolerated, imperfect conjugation results in poor focal quality, quantified by a decreased peak-to-background ratio (PBR). This need for precision poses a significant challenge when a high-quality DOPC system is constructed. Precise alignment of optical components, especially the SLM, is critical in order to measure the optimal phase map and ensure accurate mapping from the scientific complementary metal-oxide semiconductor (sCMOS) camera to the SLM for display.

DOPC may be implemented using phase-shifting holography, which requires precise alignment of optical components for an accurate phase measurement. The incident reference beam should be orthogonal to the SLM to make a uniform reference beam with both constant amplitude and phase interfere with the scattered light to maximize the period of the fringes. If the SLM is tilted or tipped out of alignment, the reference beam and, subsequently, the back-reflected sample beam, will also become misaligned, reducing the focal quality of the DOPC system. It should be noted, however, that this statement applies only to systems where the SLM is viewed by the sCMOS camera, which have become increasingly common due to their relative ease of alignment. DOPC systems in which the sCMOS camera does not view the SLM are also used, and require that the SLM be held at the same angle as the sCMOS camera with respect to the reference and sample beams, necessitating different methods of alignment and optimization.^15^^,^^28^

In order to correctly measure and conjugate the phase of scattered light through phase-shifting holography, a planar-wavefront is preferred for the reference beam. DOPC systems must, therefore, compensate for any aberration. Aberration typically arises from misalignment of and imperfection in lenses, curvature of mirrors and, most importantly, misalignment and curvature of the SLM. A compensation phase map must, therefore, be created to correct such artifacts using the SLM.^29^

In addition, the SLM itself must be carefully aligned with respect to the sCMOS camera. Because the sCMOS camera is responsible for measuring the conjugate phase map, while the SLM is used in display, mapping of the optimal phase map from one device to the other must be accurate to within a single pixel. Optimally aligning the SLM by precise adjustment of axial rotation as well as translation along the X- and Y-axes is vital to ensure pixel-to-pixel matching. Although minor misalignment may cause only translation of the focus, the PBR diminishes rapidly as the measured and displayed phase maps diverge.

Finally, as the focusing quality of the DOPC system degrades when the system drifts, frequent recalibration may be necessary to maintain the best performance of the system. A digital only compensation procedure enables a quick calibration, avoiding lengthy time-consuming iterative steps. Speed of the protocol becomes an important issue in alignment compensation methods such as those using orthonormal rectangular polynomials, which provide sufficient speed, but fail to fully correct misalignment due to their abbreviated compensation procedure.^29^

Here we present a unified protocol for optimization of a DOPC system, utilizing all digital correction for high-accuracy, high-speed compensation. By integrating look-up table (LUT) optimization, interferometry-based SLM curvature correction, and orthonormal rectangular polynomial aberration correction, our technique fully optimizes the alignment of the SLM relative to the reference beam and sCMOS camera with five degrees of freedom, while also correcting for aberration of the reference beam caused by the system’s components and SLM curvature.^30^

Five degrees of freedom are utilized by the digital alignment optimization protocol in order to allow high-speed all-digital methods to be used for all targeted corrections. Misalignment along the Z-axis is primarily minimized during the initial gross alignment using a micropositioner, as discussed in Sec. 2.2. DOPC systems have previously been shown to be much less sensitive to misalignment along the Z-axis, compared to translation along the X- and Y- axes,^28^ meaning that manual alignment of the Z-axis is sufficient to recover the majority of focal quality lost due to misalignment.

Using a digital-only compensation method based on autocovariance analysis in LUT optimization, our approach also substantially reduces the number of iterations, and therefore, the amount of time required for optimization. This fast alignment is important both for ease of use, as calibration is frequently required, and for quick measurement when the scattering medium or environmental variables cause instability in the system. In doing so, we successfully optimized our system, increasing the PBR by several orders of magnitude and allowing the system to focus through a 5-mm-thick sample with a transport scattering coefficient $\mu_{s}^{\prime }$ of $2.46\;{\mathrm{mm}}^{-1}$ at λ=500  nm, providing an optically thick diffusive medium equivalent to 12.5 transport mean free paths.

## 2 Methods

### 2.1 System Layout

**Fig. 1:**
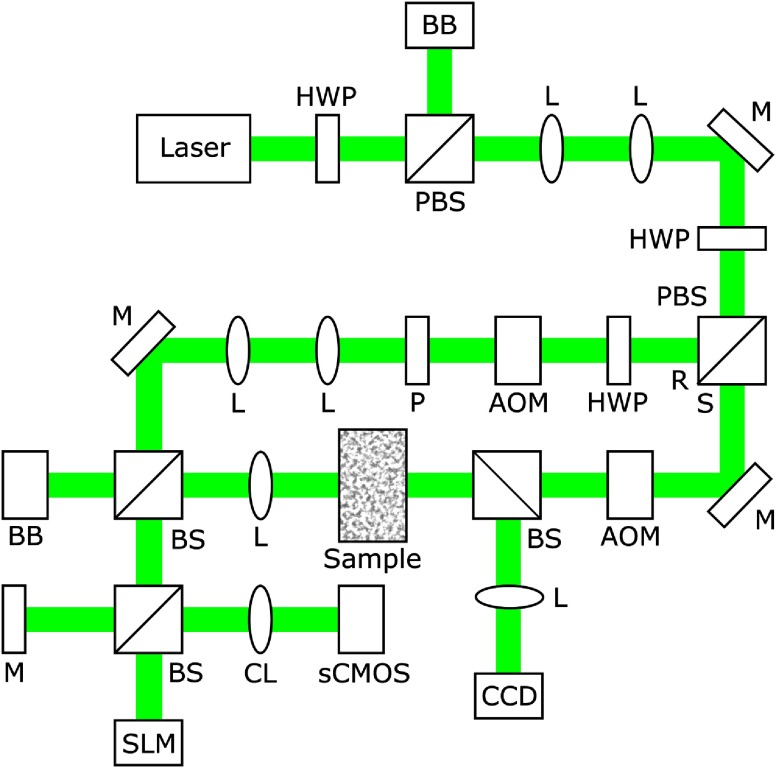
Schematic of the DOPC system. AOM, acousto-optic modulator; BB, beam block; BS, beam splitter; CCD, charge coupled device camera; CL, camera lens; HWP, half-wave plate; L, lens; M, mirror; P, polarizer; PBS, polarizing beam splitter; R, reference arm; S, sample arm; sCMOS, scientific complementary metal oxide semiconductor camera; SLM, spatial light modulator.

As shown in Fig. 1, we used an SLM (Pluto NIR-II, Holoeye) with 1920×1080 controllable elements. Illumination was provided by a 10-W continuous-wave laser (Verdi V-10, Coherent) at 532 nm wavelength. The sample and reference arms were separated by a polarizing beam splitter (PBS), with a half-wave plate prior to the PBS used to adjust the ratio of illumination to each beam. Both the reference (R) and sample (S) arms were used to acquire the optimal phase map, while a shutter blocked the sample beam in playback.

After being split, the sample beam was scattered by a diffusive medium, a 5-mm thick polyurethane phantom with uniformly dispersed titanium dioxide nanoparticles. The scattered light was then gathered by a collection lens and directed by a mirror to the SLM, where it interfered with the reference beam. The surface of the SLM was then imaged to the sCMOS camera (pco.edge 5.5, PCO) by a camera lens, which provided a demagnification of 1.23 times and matched the devices pixel-to-pixel. Because undersampling was used, the full number of controllable elements afforded by the SLM was available for optimization by the system.^31^

### 2.2 DOPC System Construction and Initial Alignment

Before implementing our alignment compensation method, gross alignment of the system must be performed in order to bring the misalignment of the system within the limits of the protocol. This manual alignment is typically required only once, with the digital alignment correction provided by the protocol sufficient to maintain alignment if performed regularly.

First, misalignment of the SLM along the Z-axis is corrected utilizing a micropositioner and interferometry provided by the mirror opposite the sCMOS camera as shown in Fig. 1. A phase pattern of 0 and π consisting of an array of crosses is displayed by the SLM. Using the micropositioner, the Z position of the SLM is then adjusted until the thickness of the lines composing the crosses are minimized, as visualized by the sCMOS camera.

After correction of translation along the Z-axis, alignment of tip and tilt must be optimized. To do so, we utilize back reflection of the laser from the mirror opposite the camera or, depending upon reflectance, the SLM. An iris is placed as early as possible within the path of the DOPC system, ideally just following the aperture of the laser, and the size of the opening minimized as much as possible while still allowing light to pass. The location of the iris is then manually adjusted to direct the minimal beam spot to the approximate center of the SLM, the angular orientation of which is adjusted to back reflect the beam to the iris using a kinematic mount with manually driven actuators.

In the case that the intensity of the beam or reflectance of the SLM is insufficient to allow for visualization of the back reflected beam at the iris, the mirror opposite the sCMOS camera may be used as a proxy for the SLM in back reflectance. If the mirror is utilized, the surface of the SLM must then be made parallel to the mirror, which may be accomplished by maximizing the period of the fringes created by interference between the mirror and SLM as visualized by the sCMOS camera.

Finally, translation misalignment of the X- and Y-axes is corrected. This alignment is carried out utilizing interferometry, this time paired with the LUT which is later optimized by the compensation protocol. A 0 and π pattern is again displayed, consisting of a series of arrows with one arrow at each of the corners of the SLM. The four corners of the SLM are then manually selected as determined by the tip of each arrow visualized by the sCMOS camera and interferometry. The coordinates of the four SLM corners are then utilized to form the LUT, with the entries mapping all other SLM pixels to the sCMOS camera pixels being calculated through interpolation of the four manually selected corners.

### 2.3 Autocovariance Analysis Look-Up Table Optimization

To ensure that the sCMOS camera and SLM are matched exactly pixel-to-pixel, an LUT is used. The LUT maps all SLM pixels to their corresponding sCMOS pixels and, in doing so, can correct misalignment of the SLM and sCMOS camera due to relative rotation or translation in the plane of the sCMOS camera sensor. The LUT also permits limited correction of affine transformation due to skew of the SLM relative to the sCMOS camera, but tilt and tip compensation of the SLM is specifically addressed later through the use of orthonormal rectangular polynomials.

**Fig. 2:**
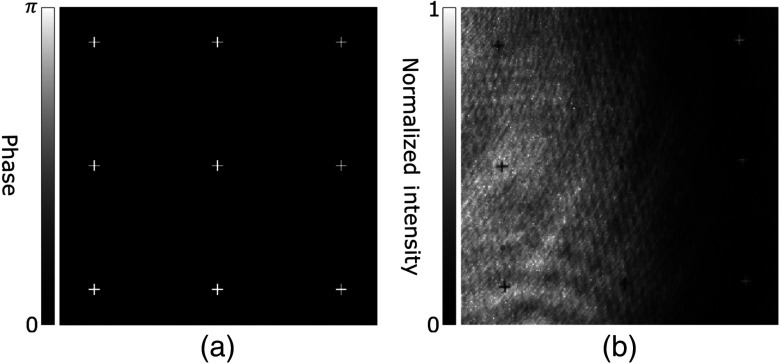
Demonstration of the expected and captured cross arrays. (a) A subsection of the displayed cross array expected at the sCMOS camera. (b) Self-normalized image of a subsection of the captured interferogram when the array of crosses is displayed on the SLM. The curvature of the SLM is manifested here by the fringes, which cause some crosses to appear black while others appear to be white.

Because the correlation time of light scattered by diffusive media depends on the stability of both the scattering medium and the DOPC system, it is important to avoid the use of time-consuming iterative methods in LUT optimization. Instead, we utilized a digital compensation method based on autocovariance analysis between a phase pattern displayed by the SLM and the pattern expected at the imaging plane of the sCMOS camera. An array of crosses, each measuring 9×9  pixels, with a phase of π and a background phase of 0 was displayed on the SLM, as seen in Fig. 2(a), which was illuminated only by the reference beam. To visualize this pattern, a Michelson interferometer was created by placing a mirror at the BS prior to the SLM, as shown in Fig. 1. To ensure accurate localization of each cross pattern, the mirror was positioned at an equal distance to the SLM from the BS, where it was also imaged by the sCMOS camera.

Once visualized by the sCMOS camera, the pattern was processed by an initial LUT derived from interpolation of the four SLM corners, as determined manually. The processed pattern was then compared to the expected pattern, i.e., the optimal pattern assuming exact 1:1 pixel matching, using autocovariance analysis of the crosses. Because the curvature of the SLM creates fringes, the imaged cross pattern is composed of crosses at both high and low intensities, as seen in Fig. 2(b). Thus when performing autocovariance analysis, the expected position of each individual cross is inspected for similarity with a cross at both the maxima or minima (see Appendix A for pseudocode).

As the LUT was adjusted, autocovariance computation quantitatively determined the similarity of the visualized pattern to the expected pattern. The corners were then iteratively moved and the accuracy of each subsequently generated LUT was evaluated using autocovariance analysis. Reaching a maximum autocovariance signals that an optimally accurate LUT had been generated, correcting for alignment errors due to relative rotation and translation in the plane of the SLM and sCMOS camera sensor.

Using autocovariance analysis to quantitatively evaluate progressively generated LUTs, we can obtain the optimal LUT without direct evaluation of the DOPC focus as feedback. This approach has several significant advantages. As a purely computational method, autocovariance analysis greatly improves the speed of LUT optimization when compared to iterative DOPC measurement. Although the LUT optimization is still iterative, removing as many iterative steps as possible from the process greatly improves the speed of the alignment compensation. Using a computational approach, we were able to optimize the LUT within tens of seconds. This speed is especially important because drift in alignment creates the need for frequent optimization of DOPC systems.

Direct evaluation of alignment using autocovariance analysis also increases the efficiency of LUT optimization by avoiding the confounding variable of decorrelation, which may arise from system instability and movement of the scattering media. Evaluation through DOPC feedback requires that DOPC foci be replicable, but decorrelation of the scattering media through which the focus is formed frequently causes variation in the measured PBR of the focus. Further variation is introduced by system instability, and when paired with the strict alignment requirements of DOPC, may result in large changes in PBR through small shifts in the system. By utilizing autocovariance analysis and iteratively generating LUTs, the optimal LUT can be obtained from a single image acquisition, eliminating potential error due to experimental variation within the runtime of the procedure.

Implementing and optimizing the LUT are vital first steps of the alignment process, because they allow a DOPC focus to be achieved without relying on feedback from the DOPC focus itself. Without a LUT and LUT optimization, it is difficult to obtain a DOPC focus. Minute misalignment may render the DOPC system unable to form a focus, and unoptimized LUTs may produce a low-quality focus.

### 2.4 SLM Substrate Curvature Correction

Curvature across the SLM substrate introduced during the manufacturing process distorts the wavefront of both the reference and sample beams. Because the system prefers a planar reference beam for accurate phase measurement, this aberration must be corrected. To do so, Michelson interferometry is again utilized. The sample beam is blocked, and only the reference beam is directed to the SLM and mirror, which form the interferometer. Using phase-shifting holography, we obtained a phase map of the SLM curvature. Four intensity measurements were made as the phase of the SLM was rotated, and the phase map was calculated by $\phi (x,y)=\arg\{[I_{0}(x,y)-I_{\pi }(x,y)]+i[I_{\frac{3\pi }{2}}(x,y)-I_{\frac{\pi }{2}}(x,y)]\}$, where arg computes the principal value of the argument of a complex number (also known as atan2) and $I_{0}$, $I_{\frac{\pi }{2}}$, $I_{\pi }$, and $I_{\frac{3\pi }{2}}$ are the recorded intensities when the phases of the SLM are 0, $\frac{\pi }{2}$, π, and $\frac{3\pi }{2}$, respectively.^21^^,^^32^ The phase map was then conjugated to create a compensation map for the SLM’s curvature. This compensation map can be used to remove the aberration of the reference beam caused by the curvature of the SLM.

### 2.5 Angular Correction Utilizing Orthonormal Rectangular Polynomials

**Fig. 3:**
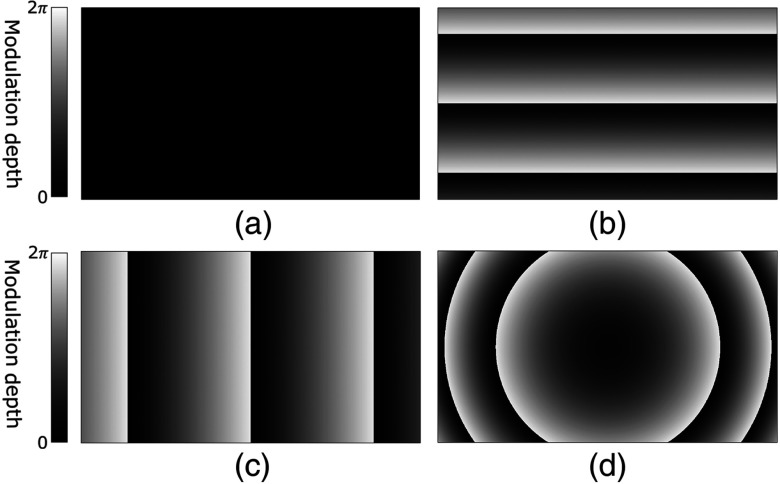
Illustration of the first four orthonormal polynomial modes used in correction of the SLM angle, and in reference beam collimation and aberration. (a) First rectangular polynomial mode, commonly referred to as “piston.” This mode is not used in calibration optimization because it serves only to digitally increase or decrease the uniform phase of the SLM. (b) Second rectangular polynomial mode, commonly referred to as “tilt.” This mode is used to digitally control the vertical angle of the SLM. (c) Third rectangular polynomial mode, commonly referred to as “tip.” This mode is used to digitally control the horizontal angle of the SLM. (d) Fourth rectangular polynomial mode, commonly referred to as “defocus.” This mode is used to digitally control the collimation of the reference beam.

Rectangular polynomials complete the alignment compensation protocol, allowing the SLM to be digitally aligned orthogonally to the sample beam. Without this alignment, minor variations in the angle of the SLM relative to the reference beam will result in an unintended phase ramp, which may significantly impact the focal quality provided by the system. To digitally adjust the angle of the SLM, rectangular polynomial modes are optimized, the second, third, and fourth of which are defined as tilt, tip, and defocus, as shown in Fig. 3.^29^^,^^30^ Application of the second and third orders corrects tilt and tip, i.e., the vertical and horizontal angles, of the SLM by creating a digital phase ramp, which is displayed on the SLM. The virtual angle of the SLM is controlled by the slope of the phase ramp, while the direction, i.e., tilt versus tip, is determined by the direction of the ramp, as shown in Figs. 3(b) and 3(c).

Higher-order polynomials may also be used to compensate for aberration introduced to the reference beam by imperfect or misaligned components in the system. Though typically less influential on the PBR of the focus, correction by the higher-order modes improves the quality of the conjugated phase map by mitigating aberration and restoring the reference beam to a plane wave. For example, the fourth mode, as seen in Fig. 3(d), digitally compensates for decollimation within the reference beam.

To optimize the rectangular polynomial, the modes are evaluated in series, with the optimal depth of modulation determined by iterative feedback with the PBR of the DOPC focus. The sensitivity of the compensation map may be determined by adjusting the number of steps when testing the optimal modulation depth for each mode. Likewise, the fidelity of compensation and aberration correction may be increased by tailoring the number of optimized modes. An increasing number of modes results in a corresponding increase in fidelity of compensation, at the cost of speed of optimization. In the same way, a larger number of steps when optimizing the depth of modulation allows for finer optimization. For this reason, we choose to optimize the first 10 modes, excluding the first which is simply piston, with a range of −10 to 10 and a step size of 0.5 for the modulation coefficients of the basis provided by the orthonormal rectangular polynomials.^30^ Utilizing these parameters, we were able to obtain an optimal phase compensation map within 1 min. Appendix A presents pseudocode and code snippet examples of the optimization process and settings described above.

### 2.6 Diffusive Optical Phantoms

To test scattering media that are stable under high-power laser illumination, a tissue mimicking phantom with a scattering coefficient comparable to that of biological tissues was fabricated. To minimize Joule heating, which would change the scattering property of the phantom, light absorption must be suppressed as much as possible. Polyurethane with titanium oxide (${\mathrm{TiO}}_{2}$, white rutile ${\mathrm{TiO}}_{2}$ powder, Atlantic Equipment Engineers) nanoparticles as a scattering additive creates a stable medium, with a correlation time greater than the runtime of our alignment compensation procedure. The polyurethane base material consists of isocyanate (part A, PX 5210) and polyol (part B, PX 523), which are liquid at room temperature. A stock solution of ${\mathrm{TiO}}_{2}$ (10  g/kg) in part A was prepared using the following procedure. Toluene (20 mL) was placed in a clean glass jar (125 mL) and 1 g of ${\mathrm{TiO}}_{2}$ was added. The solution was sonicated in a bath sonicator for 6 h until the particles were completely dispersed. Part A (100 g) was added to the same jar and sonicated in the bath sonicator for another 6 h, after which the jar was placed in a rocking mixer for at least 24 h for homogenization. The jar was then placed in a fume hood for a few days until the toluene was completely evaporated from the mixture. This stock solution was diluted with part A to obtain the final concentration of ${\mathrm{TiO}}_{2}$ in part A. For phantom fabrication, part B, in a ratio of 100 (part A):85 (part B) by weight, was added into the part $A/{\mathrm{TiO}}_{2}$ mixture, and the solution was mixed thoroughly with a blade mixer for 10 min. The final ${\mathrm{TiO}}_{2}$ concentration was 0.2% (w/w) of the total mixture, with a reduced scattering coefficient of $\approx 2.5\;{\mathrm{mm}}^{-1}$ and an absorption coefficient of $<0.01\;{\mathrm{mm}}^{-1}$ at λ=500  nm. This mixed solution was put in a vacuum desiccator to remove trapped air bubbles and prevent undesirable scattering once cured. The sample was gently poured into 6- or 10-cm diameter bacteriological Petri dishes (Falcon, Fisher Scientific) with the inside surface coated with a thin film of petroleum distillates (Mold Release 870NA Aerosol) for easy mold-release after curing. The sample was placed on a leveled platform and left overnight at room temperature for slow curing to prevent thermally driven phase separation and clustering of the scattering particles, followed by baking in an oven at 75°C for >2  h for the final curing step. The sample was then separated from the Petri dish mold for measurements. The wavelength-dependent optical properties of the 5-mm-thick phantom were measured by a single integrating sphere installed at the National Institute of Standards and Technology, and the scattering and absorption coefficients were measured by a modified inverse adding double algorithm. The experimental setup and details of the algorithms are described elsewhere.^33^

## 3 Results

After the alignment procedure, an optimized LUT and compensation phase map were obtained and applied to the SLM to achieve accurate alignment and calibration of the system. As a result, we were able to significantly increase the quality of the focus as defined by the PBR, and to focus through highly scattering thick diffusive media.

### 3.1 Contributions of LUT Optimization and SLM Curvature Correction to Focal Quality

**Fig. 4:**
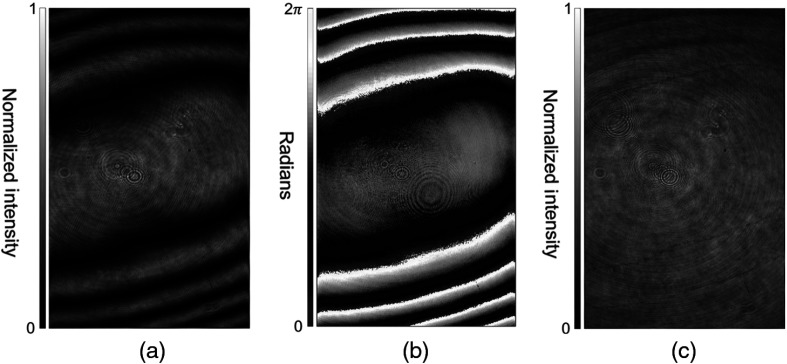
Demonstration of SLM substrate curvature correction. (a) Interferogram of initial uncompensated SLM curvature. (b) Phase map of the SLM captured through phase-shifting holography. (c) Interferogram of SLM curvature with display of the compensation phase map.

First, in order to produce a DOPC focus for testing, the LUT was optimized to correct misalignment in translation and rotation, and the SLM curvature was compensated for. As shown in Fig. 4(a), an uncorrected SLM has a large substrate curvature, covering a range >2π. Because our SLM can modulate light only over a range of 2π, this phase was wrapped and conjugated in order to obtain the compensation map, as shown in Fig. 4(b). As shown in Fig. 4(c), the corrective phase map accurately compensates for the curvature, greatly increasing the uniformity of the captured interferogram. The fringes from the SLM curvature are almost wholly removed, indicating that the phase differences in the compensated interferogram do not exceed π. Some small phase shifts can be seen, likely arising from imperfect measurement when creating the compensation phase map as well as incomplete correction fidelity owing to the digital nature of the phase measurement and display.

**Fig. 5:**
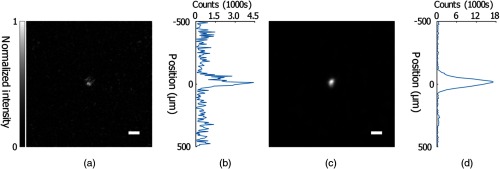
DOPC focusing through 5-mm-thick scattering sample with and without compensation of the LUT through autocovariance analysis and the SLM curvature. (a) Self-normalized image of a DOPC focus after scattering prior to optimization. PBR=15±2 (n=3). (b) Intensity profile of a vertical line crossing the peak position of the focus. (c) Self-normalized image of a DOPC focus after scattering following LUT optimization. PBR=63±1 (n=3). (d) Intensity profile of a vertical line crossing the peak position of the focus. Scale bar: 100  μm.

The performance of our optimization protocol was then quantified using the PBR, defined as the ratio of the average intensity of the focal peak, the area where intensity is greater than half the maximum intensity, to the mean intensity when a random wavefront was displayed by the SLM. As Fig. 5(a) demonstrates, the unoptimized system produces a focus with a PBR of 15±2 (n=3) when scattered by the 5-mm-thick polyurethane phantom. Figure 5(b) shows the distribution of the focal intensity in the vertical dimension, which demonstrates our focal spot size to be $3.3\times 10^{3}\pm 1.4\times 10^{2}\;\mu m^{2}$ (n=3). The speckle size was $8.2\times 10\;\mu m^{2}$ as calculated by the full width at half maximum of the autocovariance function of the speckle pattern captured by the charge coupled device camera. The maximum theoretical PBR for perfect full-aperture lossless time reversal was, therefore, $\approx 4.0\times 10^{4}$, meaning that the unoptimized system achieved about $3.7\times 10^{-2}\%$ of the theoretical maximum. After the LUT was optimized and the curvature was corrected, the focus is shown in Fig. 5(c), with a PBR of 63±1 (n=3). The vertical intensity distribution in Fig. 5(d) shows a focal spot size of $3.3\times 10^{3}\pm 0\;\mu m^{2}$ (n=3). Note that this standard deviation of zero results from the same spot size in three separate measurements. The LUT optimized system, therefore, achieved $∼1.6\times 10^{-1}\%$ of the theoretical maximum PBR. Our autocovariance analysis LUT and curvature compensation procedure, therefore, produced an improvement of ≈4 times over the original unoptimized LUT, which was aligned manually by eye with misalignments of ≈8 and 8  μm along the X- and Y-axes as well as tilt and tip of ≈76.4 and 152.8  μrad. As is clearly demonstrated, the ability of the system to focus through thick scattering media is also enhanced.

### 3.2 Orthonormal Rectangular Polynomials

Orthonormal rectangular polynomials complete the alignment compensation protocol by ensuring that the SLM surface is aligned orthogonally to the reference beam. Correcting small variations in the SLM relative to the reference beam improves the focal quality by removing unintended phase ramps.

**Fig. 6:**
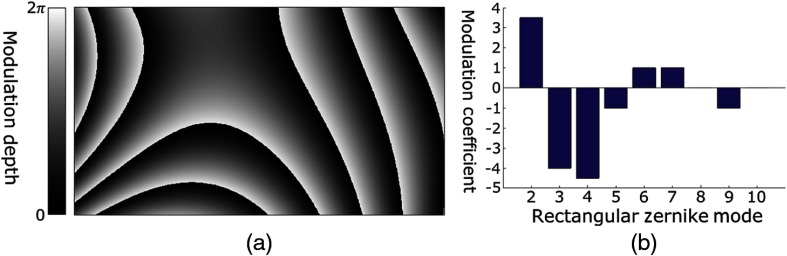
Illustration of compensation phase maps obtained using orthonormal rectangular polynomials and coefficients of contributing modes. (a) Compensation phase map for the DOPC system determined through feedback when focusing through the 5-mm polyurethane phantom. (b) Contribution of each mode to the compensation phase map seen in (a). The first mode, known as “piston” was not included as it contributes uniform adjustment of the phase and, as such, does not affect compensation.

**Fig. 7:**
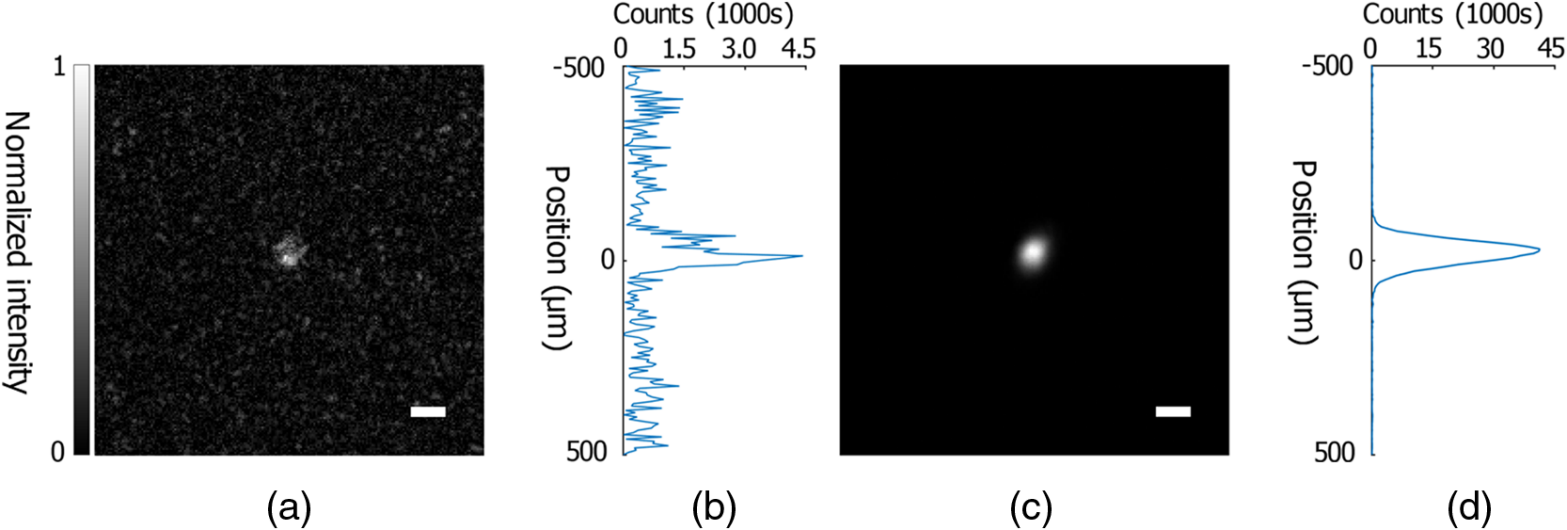
DOPC focusing through multiple scattering samples before and after complete compensation. (a) Self-normalized image of DOPC focus after scattering by 5 mm of polyurethane prior to optimization. PBR=15±2 (n=3). (b) Intensity profile of a vertical line crossing the peak position of the focus. (c) Self-normalized image of DOPC focus after scattering by 5 mm of polyurethane following optimization. PBR=2.5×103±1.6×102 (n=5). (d) Intensity profile of a vertical line crossing the peak position of the focus. Scale bar: 100  μm.

As seen in Fig. 7(a), when focusing through the 5-mm polyurethane phantom, the uncorrected system produces a focus with a PBR of 15±2. The area of the focus, as demonstrated by the vertical intensity distribution, Fig. 7(b), was $3.1\times 10^{3}\pm 1.4\times 10^{2}\;\mu m^{2}$. In comparison, with the completed alignment protocol, the PBR was $6.3\times 10^{3}\pm 1.6\times 10^{2}$, as seen in Fig. 7(c), and the area of the focal spot was $3.1\times 10^{3}\pm 9\;\mu m^{2}$ for the optimized system. The completely aligned system, therefore, achieved ∼14.6% of the theoretical maximum enhancement. This optimization represents a ≈417 times increase in PBR for the corrected versus uncorrected alignment. The compensation phase map obtained through rectangular orthonormal polynomials used for the phantom is shown in Fig. 6(a), with the first 10 modes contributing as shown in Fig. 6(b). Modes two through four contribute substantially to the alignment correction, by correcting the tilt, tip, and focus of the SLM, respectively. This dramatic improvement in PBR clearly demonstrates the utility of our protocol in allowing the DOPC system to achieve focusing in circumstances that would otherwise not allow it.

## 4 Discussion

Due to its short average mode time, i.e., the runtime of the system per optimized mode, DOPC has emerged as one of the most promising methods for focusing through scattering materials.^22^ By utilizing an sCMOS camera to globally determine the optimal phase map, DOPC’s performance is no longer restricted by the direct relationship between runtime and the optimized degrees of freedom that hinders many wavefront shaping methods, such as iterative wavefront shaping and measurement of the transmission matrix. However, the use of a camera for wavefront measurement also causes DOPC systems to be highly sensitive to misalignment and aberration, particularly of the SLM and reference beam.

Here we have presented a protocol for complete digital alignment of a DOPC system with minimal runtime. Our protocol enables correction of the DOPC system’s SLM in five degrees of freedom, vastly increasing the quality of the DOPC focus produced by the system. In addition, it corrects for aberration introduced by the SLM substrate and system components. By implementing the alignment optimization protocol, we have increased the focal quality, as quantified by PBR, by ≈417 times. In addition to improving focal quality, we were also successful in focusing through thick scattering media, as shown in Figs. 5 and 7, where the PBR was improved by ≈4 times when the LUT was corrected and the SLM curvature corrected, and by ≈417 times once the entire protocol was completed.

The alignment correction protocol was developed in our lab and has been utilized in optimizing systems for multiple studies. The procedure was used when aligning DOPC for focusing through ≈10  cm of scattering media.^32^ It was also used to improve the PBR of the world’s fastest binary and full-phase DOPC systems, at the time of publication.^8^^,^^34^ In each case, the protocol enabled large improvements in focal quality, as quantified by PBR, and, in some cases, allowed focusing through the targeted scattering medium.

It is important to note that all-digital alignment compensation can correct only relatively small errors in the DOPC system. Without frequent optimization, drift due to system instability and environmental variables may cause misalignment beyond the ability of digital methods to correct. Gross realignment of the system for an approximately optimal alignment must then be completed. For the best results, both skilled system maintenance and frequent digital alignment are crucial. Because the DOPC system must typically be optimized at least daily for the best possible performance, the high speed of the alignment compensation protocol is an important advantage in minimizing downtime.

## Appendix A: Pseudocode and Code Snippets

Here we present pseudocode and code snippets for the control algorithms utilized in our alignment compensation procedure for optimization of the LUT, in SLM substrate curvature correction through interferometry, and in SLM alignment using rectangular modes. This control code was written in MATLAB using manufacturer-supplied software development kits for control and integration of the SLM (Pluto NIR-I, Holoeye), sCMOS camera (pco.edge 5.5, PCO), and data acquisition board (NI-6321, National Instruments).

### A.1 Autocovariance Analysis Based LUT Optimization

The following pseudocode for LUT optimization assumes the Michelson interferometer is in place as shown in Fig. 1 and the sample beam is blocked as outlined in Sec. 2.3:

1. Generate a cross array pattern, with crosses having a phase of π and background having a phase of 0. Any number of crosses may be used, but the highest number possible is recommended for greater accuracy.
2. Using the Meadowlark software development kit, send the pattern phase map to SLM, and, using the sCMOS software development kit to acquire a single image.
3. Manually set an initial LUT, determined from interpolation from the four corners of the SLM as seen by the sCMOS camera.
4. Using autocovariance analysis, examine the similarity between the captured and expected patterns. Because the curvature of the SLM causes fringes within the interferogram, each cross must be individually assessed. Each captured cross is compared to the expected cross location, with both black and white crosses examined in order to account for crosses at both maxima and minima within the captured interferogram.

*function [t_xb, t_xw, t_yb, t_yw]=ACA(acqImg, lutX, lutY, expectedCross)*

 *t_xb = 0;*

 *t_xw = 0;*

 *t_yb = 0;*

 *t_yw = 0;*

 *for ii = 1:count*

  *isoCross = acqImg([lutY(a(ii),b(ii))*-

   *10:lutY(a(ii),b(ii))+10],[lutX(a(ii),b(ii))-10:lutX(a(ii),b(ii))+10]);*

   *%Isolates the area about the %expected location of each cross, with “a” and*

   *“b” %corresponding to the X and Y positions of the generated %crosses.*

  *isoCross_b = max(max(isoCross))-isoCross; %Represents black %cross*

  *isoCross_w = isoCross-min(min(isoCross)); %Represents white %cross*

  *YY_b = conv2(isCross_b,expectedCross); %Convolve expected and %acquired crosses.*

  *YY_w = conv2(isoCross_w,expectedCross);*

  *[dx_b(ii),dy_b(ii)] = find(YY_b == max(max(YY_b)),1); %Find X %and Y coordinates of the maximum value.*

  *dx_b(ii) = dx_b(ii)-15; %Normalize the maximum indices based on %expected position. Note: This value will change based on the %size of the isolated are for the acquired cross.*

  *dy_b(ii) = dy_b(ii)-15;*

  *[dx_w(ii),dy_w(ii)] = find(YY_w == max(max(YY_w)),1);*

  *dx_w(ii) = dx_w(ii)-15;*

  *dy_w(ii) = dy_w(ii)-15;*

  *%Quantify the number of crosses at the expected positions.*

  *if dx_b(ii)==0*

   *t_xb = t_xb+1;*

  *end*

  *if dx_w(ii)==0*

   *t_xw = t_xw+1;*

  *end*

  *if dy_b(ii)==0*

   *t_yb = t_yb+1;*

  *end*

  *if dy_w(ii)==0*

   *t_yw = t_yw+1;*

  *end*

 *end*

*end*

5. Iteratively move each corner of the LUT within a set range. For each new corner position, assess the similarity of the expected and acquired pattern and record.

*for pt1 = -test_range:test_range*

 *for pt2 = -test_range:test_range*

  *for pt3 = -test_range:test_range*

   *for pt4 = -test_range:test_range*

    *%Set corners for LUT*

    *x1_trial = x1+pt1;*

    *x2_trial = x2+pt2;*

    *x3_trial = x3+pt3;*

    *x4_trial = x4+pt4;*

    *y1_trial = y1+pt1;*

    *y2_trial = y2+pt2;*

    *y3_trial = y3+pt3;*

    *y4_trial = y4+pt4;*

    *%Construct LUT*

    *row_begin = linspace(x1_trial,x3_trial,N);*

    *row_end = linspace(x2_trial,x4_trial,N);*

    *column_begin = linspace(y1_trial,y2_trial,M);*

    *column_end = linspace(y3_trial,y4_trial,M);*

    *X=zeros(N,M);*

    *Y=zeros(N,M);*

    *for ii = 1:N*

     *X(ii,:) = linspace(row_begin(ii),row_end(ii),M);*

    *end*

    *for ii = 1:M*

     *Y(:,ii) = linspace(column_begin(ii),column_end(ii),N);*

    *end*

    *X = round(X);*

    *Y = round(Y);*

    *[t_xb, t_xw, t_yb, t_yw]=ACA(acqImg, lutX, lutY, expectedCross) %Run ACA*

    *%Track results of* *autocovariance analysis* *for each corner point and*

    *%modify.*

    *if (t_xb+t_xw) > max_x*

     *y1_max = y1_trial;*

     *y2_max = y2_trial;*

     *y3_max = y3_trial;*

     *y4_max = y4_trial;*

     *max_x = t_xb+y_xw;*

     *max_x_b = t_xb;*

     *max_x_w = t_xw;*

    *end*

    *if (count_y_b+count_y_w)> max_y*

     *x1_max = x1_trial;*

     *x2_max = x2_trial;*

     *x3_max = x3_trial;*

     *x4_max = x4_trial;*

     *max_y = count_y_b+count_y_w;*

     *max_y_b = count_y_b;*

     *max_y_w = count_y_w;*

    *end*

   *end*

  *end*

 *end*

*end*

6. Once a maximum is reached through autocovariance analysis, an optimal LUT has been acquired. Autocovariance analysis similarity should be evaluated for crosses at both maxima and minima combined.

### A.2 SLM Curvature Correction

The pseudocode for SLM substrate curvature correction is as follows, with the Michelson interferometer in place as shown in Fig. 1:

1. Rotate the phase of SLM through 0, π/2, π, and 3 π/2.
2. At each phase, acquire a single image using the sCMOS camera.
3. From the four captured images, given as $I_{p}(x,y)$, where p is the set phase of the SLM, the phase of the SLM can be calculated as $\phi (x,y)=\arg\{[I_{0}(x,y)-I_{\pi }(x,y)]+i[I_{\frac{3\pi }{2}}(x,y)-I_{\frac{\pi }{2}}(x,y)]\}$, where arg computes the principal value of the argument of a complex number (known also as atan2).
4. Once the phase map at the SLM has been calculated, the compensation phase map is obtained through conjugation.

### A.3 Orthonormal Rectangular Polynomial Aberration and Angular Compensation

The pseudocode for orthonormal rectangular polynomial optimization is as follows, using feedback from the DOPC foci:

1. Generate baseline patterns for lower order rectangular polynomials.

*SLMx = 1080; %SLM X dimension.*

*SLMy = 1920; %SLM Y dimension.*

*Orders = 10; %Number of orders to be used. For this example, 10 are shown.*

*%Normalization terms for unit rectangle.*

*a = 1080/sqrt(SLMx^2+SLMy^2);*

*xmax = a;*

*ymax = sqrt(1-a^2);*

*x = linspace(-xmax,xmax,M);*

*y = linspace(-ymax,ymax,N);*

*rPolynomials = zeros(SLMx,SLMy,orders); %Array for polynomials.*

*for ii = 1:M*

 *for jj = 1:N*

  *rPolynomials(ii,jj,1) = 1;*

  *rPolynomials(ii,jj,2) = sqrt(3)/a*x(ii);*

  *rPolynomials(ii,jj,3) = sqrt(3/(1-a^2))*y(jj);*

  *rPolynomials(ii,jj,4) = sqrt(5)/(2*sqrt(1-2*a^2+2*a^4))*(3*(x(ii)^2+y(jj)^2)-1);*

  *rPolynomials(ii,jj,5) = 3/(a*sqrt(1-a^2))*x(ii)*y(jj);*

  *rPolynomials(ii,jj,6) = sqrt(5)/(2*a^2*(1-a^2)*sqrt(1-2*a^2+2*a^4))*(3*(1-a^2)^2*x(ii)^2-3*a^4*y(jj)^2-a^2*(1-3*a^2+2*a^4));*

  *rPolynomials(ii,jj,7) = sqrt(21)/(2*sqrt(27-81*a^2+116*a^4-62*a^6))*(15*(x(ii)^2+y(jj)^2)-9+4*a^2)*y(jj);*

  *rPolynomials(ii,jj,8) = sqrt(21)/(2*a*sqrt(35-70*a^2+62*a^4))*(15*(x(ii)^2+y(jj)^2)-5-4*a^2)*x(ii);*

  *rPolynomials(ii,jj,9) = sqrt(5)*sqrt((27-54*a^2+62*a^4)/(1-a^2))/(2*a^2*(27-81*a^2+116*a^4-62*a^6))*(27*(1-a^2)^2*x(ii)^2-35*a^4*y(jj)^2-a^2*(9-39*a^2+30*a^4))*y(jj);*

  *rPolynomials(ii,jj,10) = sqrt(5)/(2*a^3*(1-a^2)*sqrt(35-70*a^2+62*a^4))*(35*(1-a^2)^2*x(ii)^2-27*a^4*y(jj)^2-a^2*(21-51*a^2+30*a^4))*x(ii);*

 *end*

end

2. Set number of iterative steps and range of modulation. Note: this will determine the depth of the displayed Zernike modes and the granularity of the iterative optimization.
3. Using the PCO sCMOS software development kit, acquire four images as the phase is rotated through 0, $\frac{\pi }{2}$, π, and $\frac{3\pi }{2}$.
4. Calculate baseline phase map from images, using phase shifting holography.
5. Send calculated phase map to SLM for display, using software development kit, and measure initial PBR.
6. Based on parameters set in Step 2, iteratively test each rectangular polynomial mode for the given range of modulation.

*compPMap=zeros(SLMx, SLMy);*

*for zMode=1:orders*

 *for modDepth= -range:2*range/(numSteps-1):range*

  *zMap=mod(zModeMaps(:,:,zMode),2*pi); %Phase map is modulated by 2π for phase wrapping.*

  *testPMap=mod((compPMap+basicPMap+zMap),2*pi)/(2*pi)*255; %255%is used as the SLM provides a 8 bit controller.*

  *dispSLM(testPMap); %Display test phase map on SLM.*

  *PBR=measPBR(imgCap); %Quantify PBR of acquired focus.*

  *if(PBR>optPBR)*

   *optPBR=PBR;*

   *compPMap=mod(compPMap+zMap,2*pi);*

  *end*

 *end*

*end*
